# Timely initiation of breastfeeding and associated factors among mothers of infants age 0–6 months old in Bahir Dar City, Northwest, Ethiopia, 2017: a community based cross-sectional study

**DOI:** 10.1186/s13006-018-0196-3

**Published:** 2019-01-14

**Authors:** Amare Belachew

**Affiliations:** College of Medicine and Health Sciences, Bahir Dar University, Bahir Dar, Ethiopia

**Keywords:** Timely initiation breastfeeding, Infants less than six months, Bahir Dar, Ethiopia

## Abstract

**Background:**

Early initiation of breastfeeding, also known as early initiation, is the provision of a mothers own breast milk to her infant within one hour of birth. In Ethiopia, there is a considerable variation in the timely initiation of breastfeeding practices. Therefore, the objective of this study was to assess the level of timely initiation of breastfeeding and associated factors among mothers of infants less than six months old in Bahir Dar, Northwest, Ethiopia.

**Methods:**

A community-based cross-sectional study was conducted in Bahir Dar City from April 15 to May 3, 2017. A total of 472 mothers of infant age less than six months were selected by simple random sampling technique. Data were collected using an interviewer administered questionnaire. Descriptive statistics were done to know the distribution of variables. To identify predictors logistic regression was conducted.

**Results:**

The prevalence of timely initiation of breastfeeding was 356 (75.4%). Mothers who birth by a vaginal delivery (Adjusted Odds Ratio [AOR] 6.99; 95% Confidence Interval [CI] 3.49, 14.00), mothers who gave birth at health institution (AOR 3.36; 95% CI 1.47, 7.67), and who get breastfeeding counseling during antenatal care visits (AOR 5.64; 95% CI 2.70, 11.79) were more likely to initiate breastfeeding within one hour than counterparts.

**Conclusions:**

Practice of timely initiation of breastfeeding in this study was suboptimal. Mothers who delivered at the health institution, gave birth by a vaginal delivery, and who got breastfeeding counseling during antenatal visits were the independent predictors of the timely initiation of breastfeeding practice. Encouraging all mothers to give birth in health facilities, counsel mothers to initiate breastfeeding timely at time of Caesarean sections, reduce the indication of the Caesarean procedure and providing breastfeeding counseling during antenatal care visits were recommended. Additionally, health services must establish practices that enable timely breastfeeding whenever possible, in particular, after Caesarean section and systems need to be set up to enable skin-to-skin and timely breastfeeding.

## Background

Breastfeeding is important for the growth and development of newborn babies by providing vital nutrients. World Health Organization (WHO) recommend that breastfeeding should be initiated within the first hours of life and exclusive breastfeeding is enough, without any additional foods or drinks up to six months [1]. Breastfeeding helps to increase bonding between mother and infant, reduce infections like pneumonia and diarrhea, enhance growth and development of infants, and reduce the risk for obesity in later life [2].

Timely initiation of breastfeeding can help to prevent neonatal deaths caused by infections such as sepsis, pneumonia, and diarrhea. The study showed that when breastfeeding was initiated within the first hour, around 22% of neonatal mortality could be prevented [3].

Other studies were done on timely initiation and exclusive breastfeeding practices in many parts of the world, including in Ethiopia. Based on Ethiopian Demographic and Health Survey (EDHS) 2016 report, only 73% mothers started breastfeeding within one hour of birth [4]. Similarly, studies done in Motta town, Easten Gojjam zone 78.8%, Debre Birhan town 62.6% and Dembecha district 73.1% showed that infants started breastfeeding within one hour of birth respectively [5–7].

Studies done in Ethiopia showed that mothers when gave birth by traditional birth attendants, place of delivery was at home, didn’t provide colostrum within the first hour, had inadequate information regarding breastfeeding practice and prelacteal feeding, could have an influence on timely initiation and exclusive breastfeeding practices [5, 8, 9]. In Ethiopia, there is a considerable variation of prevalence in timely initiation of breastfeeding practices and this has not achieved the plan set at Ethiopian Health Sector Development Plan (HSDP) IV, which is to increase in the proportion of timely initiation of breastfeeding to 92% by the end of 2015 [10]. Therefore, the aim of this study was to assess the prevalence of timely initiation of breastfeeding and associated factors among mothers of infant age 0–6 months old in Bahir Dar City, Ethiopia.

## Methods

### Study area and design

A community-based cross-sectional study was employed from April 15 to May 3, 2017. The study was conducted in Bahir Dar City, which is the capital city of Amhara, a national regional state, Ethiopia. It is 565 km away from Addis Ababa. The city has nine sub cities and with a total of 17 kebeles. The total population of Bahir Dar City was 308,877. Of this population 151,350 (49%) were males and 157,527 (51%) were females. The city has government and private health institutions. It has two government and private hospitals, and nine health centers.

### Sampling size and sampling procedure

The sample size was calculated using single proportion formula with the assumption of 95% confidence level, 5% margin of error, 82% proportion of timely initiated breastfeeding practice in Ambo [11] and using design effect of two, then 10% non-response rate was added and finally it became 499. Since it is multistage (among nine sub-cities, six sub-cities were selected and then kebeles in selected sub cities was selected by lottery methods). The total sample size was proportionally allocated in each selected kebeles. Simple random sampling i.e., lottery method was used to select study participants. Eligible mothers of having an infant less than six months old were traced from health extension workers. The actual age of the infants was determined by asking the mothers and reviewing the birth date certificate.

### Data measures

An interviewer administered questionnaire was used to collect the data. The questionnaire was developed from reviewing from previous research done on a similar topic [4–7]. Data collectors and supervisors were trained on how to extract information from respondents, ethical aspects and ways of communication one-day training prior to study commencement.

### Statistical analysis

The questionnaire was checked visually for completeness and coding was given at the right margin of the questionnaire. Data was entered into Epi info version 3.5.3 and exported to SPSS version 21.0 statistical software packages for analysis. Crude (COR) and adjusted odds ratio (ARO) with 95% confidence interval (CI) was calculated to determine the strength of association between a response variable and predictor variables. Variable with a *p* value less than 0.05 was declared as a level of significance.

### Variables

#### Dependent variables

Timely initiation of breastfeeding practice.

#### Independent variables

Sociodemographic characteristics of mother’s i.e. income, education, religion, marital status, family type, age, ethnicity, occupation, information etc.

Healthcare service use by the mother’s e.g. number of antenatal visits, mode of delivery, place of delivery, breastfeeding counseling during antenatal visits, prelacteal feeding, colostrum feeding etc.

Sociodemographic characteristics of infants i.e. gender of newborn etc.

Timely (early) initiation of a breastfeeding: a mother who put her baby to breast within one hour following delivery [5].

Prelacteal feeding: an infant within the first three days of life being fed something other than breast milk [5].

Colostrum feeding: mother provides the first breast milk which is thick, sticky and clear yellowish in color contains (maternal antibody, vitamin A, protein) to their infant within one hour [1].

## Results

### Sociodemographic characteristics

Among 499 eligible mothers, 472 were agreed to participate in this study, which made a response rate of 94.5%. Regarding infant gender, around 45.3% of children were males and 54.7% of children were females. The majority 261 (55.3%) of children were aged between four to six months. The mean age of mothers was 27.5 years, 163 (34.5%) were within the age group of 25–29 years. One hundred and eight (22.9%) mothers had attended primary education and 53.4% were housewife mothers. From 472 mothers, 298 (63.1%) were living in a nuclear family. The average house income of the respondents was 1750 Ethiopian birr per month and 59 (12.5%) respondents earn less than or equal to 1000 birr per month (Table 1).

### Breastfeeding related practices

All mothers have breastfed their current infant for a period of time. From a total of 472 mothers, 356 (75.4%) initiated breastfeeding within one hour of birth. Most mothers 396 (83.9%) fed colostrum/first milk to their newborn. The majority, 74.6% did not give a prelacteal food other than breast milk within three days of an infant life. The prevalence of exclusive breastfeeding practice one day before the survey was 86.4% (Table 2).

**Table 1:** Sociodemographic characteristics of mothers (respondents) who have infants less than six months old, in Bahir Dar town, Northwest, Ethiopia, 2017

Variable	Category (*n* = 472)	Frequency	Percent
Infant sex	Male	214	45.3
	Female	258	54.7
Infant age	0–30 days	71	15
	31–90 days	140	29.7
	91–180 days	261	55.3
Age of mothers	15–19	30	6.4
	20–24	119	25.2
	25–29	163	34.5
	30–34	98	20.8
	35 and above	62	13.1
Religion	Orthodox	355	75.2
	Muslim	94	20
	Protestant	12	2.5
	Catholic	11	2.3
Ethnicity	Amhara	455	96.4
	Tigre	6	1.3
	Oromo	6	1.3
	Others	5	1
Level of education of mothers	No education	86	18.2
	Able to read & write	55	11.7
	Primary school (1–8)	108	22.9
	Secondary	86	18.2
	Higher	137	29
Occupational status of mothers	Housewife	252	53.4
	Governmental employee	108	22.9
	Private employee	46	9.7
	Merchant	38	8.1
	Daily labor	28	5.9
Marital status of mother	single	36	7.6
	Married	409	86.7
	Widowed	6	1.3
	Divorced	21	4.4
Husband educational level (409)	No education	30	7.3
	Able to read & write	51	12.5
	Primary school (1–8)	103	25.1
	Secondary	85	20.80
	Higher	140	34.3
Husband occupation (409)	Gov.t employee	118	28.9
	Private employee	77	18.8
	Merchant	116	28.4
	Daily labor	64	15.6
	Farmer & driver	34	8.3
Type of family	Nuclear family	298	63.1
	Extended family	174	36.9
Household income	≤ 1000	59	12.5
	1001–2000	160	33.9
	2001–3000	62	13.1
	> 3000	191	40.5

**Table 2:** Breastfeeding and related practice among mothers who have an infant less than six months in Bahir Dar City, Northwest, Ethiopia, 2017

Variable	Category	Frequency	Percent
Antenatal visits	Yes	370	78.4
	No	102	21.6
Number of antenatal visits (370)	≤ 3	219	59
	≥ 4	151	41
Breastfeeding counseling during antenatal visits (370)	Yes	203	54.9
	No	167	45.1
Place of delivery	Home	119	25.2
	Health institution	353	74.8
Mode of delivery	Vaginal	287	60.8
	Caesarean section	185	39.2
Breastfeeding experience of current infant	Yes	461	97.7
	No	11	2.3
Timely initiation of breastfeeding within 1 h	yes	356	75.4
	No	116	24.6
Colostrum feeding	Yes	396	83.9
	No	76	16.1
Prelacteal feeding	Yes	120	25.4
	No	352	74.6
Infant feeding in the previous 24 h.	Exclusively BF	408	86.4
	In addition to breast milk water, sugar solution, tea, soft foods.	64	13.6
Reasons for not exclusively BF (64)	Not sufficient for infant	19	29.7
	culture/tradition	17	26.7
	Lack of time	12	18.7
	Maternal illness	9	14.1
	Infant becomes thirsty	5	7.8
	Nipple breast problem	2	3

**Table 3:** Factors that affect timely initiation of breastfeeding practice among mothers of infants aged less than 6 months using bivariate and multivariate logistic regression analysis, Bahir Dar, Ethiopia, 2017

Variables	Timely initiation of breastfeeding				
		Yes *n* (%)	No *n* (%)	Crude Odds Ratio (95% CI)	Adjusted Odds Ratio (95% CI)
Place of delivery	Home	79 (66.4)	40 (33.6)	1	1
	Health institution	276 (78.2)	77 (21.8)	1.81 (1.019,2.681)	3.36 (1.476, 7.672)*
Mode of delivery	Vaginal delivery	225 (78.4)	62 (21.6)	1.46 (1.041, 2.522)	6.99 (3.496, 14.007)*
	Caesarean section	132 (71.4)	53 (28.6)	1	1
Occupational status	Unemployed	209 (82.9)	43 (17.1)	2.4 1 (1.272, 3.095)	2.26 (0.998, 4.310)
	Employed	147 (71)	73 (29)	1	1
Mother’s educational status	Uneducated	99 (70.2)	42 (29.8)	1	1
	Educated	257 (77.6)	74 (22.4)	1.47 (1.041, 2.609)	1.91 (0993, 3.708)
Maternal age	15-29 yrs	220 (70.5)	92 (29.5)	1	1
	≥ 30 yrs	130 (81.2)	30 (18.8)	1.81 (1.168, 3.014)	1.49 (0.764, 2.926)
Breastfeeding counseling during antenatal visit	Yes	176 (86.7)	27 (13.3)	1.85 (1.069, 3.187)	5.64 (2.702, 11.798)*
	No	130 (77.8)	37 (22.2)	1	1

1 = reference * = *p* - value less than 0.05 *n* = number % = percent

### Factors associated with timely initiation of breastfeed practice

First variables tested by using bivariate analysis and in bivariate analysis variables with (*p* < 0.05) is considered into multivariate logistic regression analysis:

In bivariate analysis the factors found to be associated with initiation of breastfeeding within one hour were; place of delivery, mode of delivery, occupational status, mother’s educational status, maternal age and breastfeeding counseling during antenatal visits.

Finally, mode of delivery, place of delivery, and counseling of breastfeeding during antenatal visits were identified as predictor variables in multivariate analysis.

Mode of delivery were significantly associated with timely initiating of breastfeeding. The odds of timely initiation were 6.9 times higher for women giving birth vaginally than those who had a caesarian section (AOR 6.9, 95% CI 3.49, 14.00).

Place of delivery was significantly associated with timely initiation of breastfeeding practices. Mothers who give birth at a health institution were 3.4 times more likely to initiate breastfeeding within one hour than mothers who delivered at home (AOR 3.36, 95% CI 1.47, 7.67).

Counseling of mothers during antenatal care visits was significantly associated with timely initiation of breastfeeding. Mothers who were counseled during antenatal visits were 5.6 times more likely to initiate breast milk within one hour than mothers who had not counseled during antenatal visits (AOR 5.64, 95% CI 2.70, 11.79) (Table 3).

## Discussion

Timely initiation of breastfeeding is vital for the survival of infants. In spite of this importance, the practice of timely initiation of breastfeeding in the study area was not satisfactory. Only 75.4% of mothers initiated timely breast milk, which is lower than the Ethiopian HSDP IV target level; to increase breastfeeding within the first hour of life from 52.1 to 92% by 2015 [10]. This rate in the southern part of Ethiopia is 83.7% [12, 13]. This finding is comparable with studies done in Motta (78.8%) [5], Bangladesh 71% [14], EDHS 73% [4] and Dembecha district, 73.1% [7]. However, this finding is higher than studies done in Arba Minch 57.6% [15], Tanzania 51% [16], and Bangladesh 67.3% [17]. The difference may be due to maternal sociodemographic characteristics like i.e. access to information, socio-economic status, infrastructure, educational status, cross cultural difference in breastfeeding practice, and health service utilization characteristics.

Place of delivery was significantly associated with timely initiation of breastfeeding in the study area. A mother who gave birth at a health institution was 3.4 times more likely to initiate timely breastfeeding than when delivered at home. This finding is similar to a study done in Motta [5], Debre Birhan [6], Bangladesh [17], Axum [9], Uganda [18] and Nepal [19]. Mothers who delivered at a health institution may be supported and made aware of importance of early initiation of breastfeeding, and also health professionals tend to facilitate early initiation of breastfeeding compared to those who gave birth at home. Another possible explanation is mothers who delivered at home more commonly practiced prelacteal feeding and this may lead to a delay in initiation of breastfeeding which is supported by research done in Ethiopia, which revealed that mothers who gave birth at home were more likely to practice prelacteal feeding than their counterparts [8, 20]. Therefore, counseling of mothers about the place of delivery during the antenatal visit, to give birth at a health institution, is crucial to optimize early initiation of breastfeeding.

Modes of delivery were also significantly associated with timely initiation of breastfeeding. A mother who gave birth via vaginal delivery was around seven times more likely to initiate breastfeeding within one hour than those gave birth via cesarean section. This finding is in line with studies done in Motta [5] and a systematic review done of Medline LILACS Scopus and Web of Science electronic databases [21]. This may be due to the procedure taking longer, pain after procedure, effects of anesthesia and tiredness that make it difficult to initiate breastfeeding early. It may be also health professionals may be busy with lifesaving activities and lack attention to initiate breastfeeding early. Therefore, health professionals and pregnant mothers could be informed about the negative association between a caesarian delivery and breastfeeding and its impact on infant wellbeing, as well as changing hospital policies and practices which are also necessary.

In this study, mothers who were counseled during antenatal visits were 5.6 times more likely to initiate timely breastfeeding than those who were not counseled during antenatal visits. This finding is consistent with studies done in Ethiopia; Motta [5], Raya Kobo [8], and Axum [9]. This may be due to mothers who had antenatal visits receive enough information about the importance of early initiation of breastfeeding and have also adequate knowledge about breastfeeding, which may lead to early initiation of breastfeeding compared to counterparts.

## Conclusion

Prevalence of timely initiation of breastfeeding in the study area was lower than the national recommended level. Place of delivery, mode of delivery and breastfeeding counseling during antenatal visits were the determinant factors for timely initiation of breastfeeding practices. Community based breastfeeding education about timely initiation of breastfeeding to pregnant women, encouraging all mothers to give birth in health facilities, counseling mothers to initiate breastfeeding timely at the time of caesarian sections and providing breastfeeding counseling during antenatal visits are recommended to increase timely initiation of breastfeeding. Additionally, health services must establish practices that enable timely breastfeeding whenever possible, particularly after Caesarean a section and systems need to be set up to enable skin-to-skin and timely breastfeeding.

## Abbreviations

ANC: Antenatal care service
C/S: Caesarean section
EBF: Exclusive Breast Feeding
EDHS: Ethiopian Demographic and Health Survey
HSDP: Health sector development plan
WHO: World Health Organization
